# Supplementary material for: Causal Relationship between Aging and Anorexia Nervosa: A White-Matter-Microstructure-Mediated Mendelian Randomization Analysis
Source: Biomedicines. 2024 Aug 16;12(8):1874. doi: 10.3390/biomedicines12081874 (PMC11351342; doi:10.3390/biomedicines12081874)
Supplement: Supplementary file 1 [file biomedicines-12-01874-s001.zip › Supplementary Figure/Figure S8/Leaveoneout plot of MR analysis for TL to ubm-b-1986.pdf]

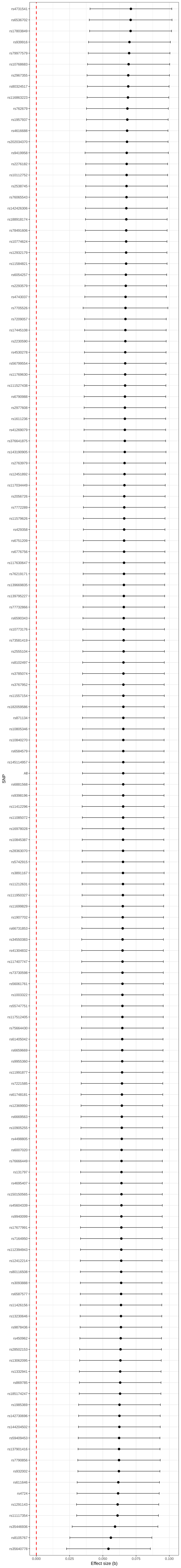
rs4731541
rs6536702
rs17803849
rs939916
rs79977579
rs10768683
rs2967355
rs80324517
rs116863223
rs762679
rs1957937
rs4616688
rs202034370
rs9419958
rs2276182
rs10112752
rs2538745
rs76065543
rs142426306
rs188918174
rs78491606
rs10774624
rs12932179
rs11584821
rs6054257
rs2293579
rs4743037
rs7705526
rs7209057
rs17445108
rs2230590
rs4530278
rs56799554
rs11769630
rs111527438
rs6790988
rs2977608
rs1611236
rs41269079
rs376641875
rs143190905
rs2763979
rs12451892
rs117034449
rs2056726
rs7772289
rs11579626
rs429358
rs6751209
rs6776756
rs117630647
rs76219171
rs139669835
rs139795227
rs77732866
rs6590343
rs10773176
rs73581419
rs2555104
rs8102497
rs3785074
rs3767952
rs11557154
rs182059586
rs871134
rs10805346
rs10840270
rs6584579
rs145114957
All
rs6881568
rs9398196
rs11412296
rs11085072
rs16978028
rs10845387
rs28363070
rs5742915
rs3891167
rs11212631
rs111950327
rs11699829
rs1907702
rs66731853
rs34550383
rs41304832
rs117407747
rs73730598
rs56061761
rs1003322
rs55747751
rs117512405
rs75664430
rs61405042
rs6659669
rs9955360
rs11991877
rs7221585
rs61748181
rs12369950
rs6669563
rs10905255
rs4498805
rs6007020
rs76666449
rs131797
rs4695407
rs150150565
rs45604339
rs9940099
rs17677991
rs7164950
rs112394943
rs12412214
rs80116508
rs3093888
rs6587577
rs11426156
rs13230646
rs9878436
rs450962
rs28502153
rs13062095
rs1332941
rs869785
rs185174247
rs1985369
rs142730696
rs144204502
rs59409453
rs137901416
rs7790856
rs932002
rs611646
rs4724
rs1291143
rs11117354
rs35446936
rs8105767
rs35640778
SNP
0.000
0.025
0.050
0.075
0.100
Effect size (b)
